# Association between serum C-reactive protein (CRP) and Omicron variant COVID-19 pneumonia in cancer patients: A multicenter cross-sectional study at the end of 2022 in China

**DOI:** 10.1097/MD.0000000000036965

**Published:** 2024-01-12

**Authors:** Kaijun Che, Zhimin Zeng, Chen Hong, Duanyang Peng, Anwen Liu, Yanqing He

**Affiliations:** a Department of Oncology, The First People’s Hospital of Fuzhou, Fuzhou, Jiangxi Province, PR China; b Department of Oncology, The Second Affiliated Hospital of Nanchang University, Nanchang, Jiangxi Province, PR China; c Jiangxi Key Laboratory of Clinical Translational Cancer Research, Nanchang, Jiangxi Province, PR China; d Radiation Induced Heart Damage Institute of Nanchang University, Nanchang, Jiangxi Province, PR China; e Department of Nosocomial Infection Control, The Second Affiliated Hospital of Nanchang University, Nanchang, Jiangxi Province, PR China.

**Keywords:** cancer patients, COVID-19 pneumonia, CRP, Omicron variants

## Abstract

Cancer patients with COVID-19 have a higher infection rate and mortality rate than non-cancer patients. However, there are few studies on the correlation between the serum C-reactive protein (CRP) and cancer patients with COVID-19. This study aims to investigate the association between serum CRP and the incidence of COVID-19 pneumonia in cancer patients at the end of 2022 in China. This cross-sectional study with a retrospective cohort between December 2022 and February 2023 assessed cancer patients complicated with COVID-19 infection in 2 Chinese institutions. Logistic regression analyses were used to compute Odds ratio (OR) and 95%CIs for the association between serum CRP and the incidence of COVID-19 pneumonia in cancer patients. A total of 213 cancer patients with COVID-19 were enrolled. Eighty-six patients (40.4%) developed COVID-19 pneumonia, among which 23 patients (10.8%) progressed to severe cases. Univariate Logistic regression showed that high CRP levels were found to be an unfavorable predictor of COVID-19 outcomes (OR = 17.9, 95%CI: 7.3, 43.6; *P <* .001). In the multivariate analysis, high CRP levels were associated with a higher incidence rate of COVID-19 pneumonia (OR = 9.8, 95%CI: 2.2, 43.8; *P* = .003). In the multivariate logistic regression model and smooth curve fitting, we found a correlation between CRP and COVID-19 pneumonia. The serum CRP was associated with the incidence of Omicron variant COVID- 19 pneumonia in cancer patients. Hence, cancer patients with high CRP level maybe need for timely computer tomography examination and more aggressive treatment.

## 1. Introduction

The Omicron variants of severe acute respiratory syndrome coronavirus 2 (SARS-CoV-2) has caused a rapid global spread since its initial detection in November 2021, exhibiting significant differences from previous strains in terms of its epidemiological characteristics, genomics, and biological behavior.^[1]^ Its affinity is 13 times that of the original strain and 2.8 times that of the Delta variant. However, its severity rate and case fatality rate are relatively low.^[2]^ The Omicron variants (BA.2 and BA.5) have presented a major challenge to Chinese COVID-19 prevention and control policies due to its unpredictable course and persistent spread. Given that Chinese government has optimized and implemented scientifically controlled epidemic prevention strategies since December 7, 2022, a large number of infections have occurred during a short period of time, and has placed a significant burden on healthcare resources.^[3–5]^ Although most of these infections are mild, the impact of Omicron variant infections on Chinese cancer patients remains not well characterized.

Cytokine storm is a major cause of mortality in severe cases of COVID-19, and Prolonged neutropenia and T-cell deficiency (lymphopenia) or dysfunction are the high risks for progression of COVID-19.^[6]^ Previous studies have found that neutrophil-lymphocyte ratio (NLR), interleukins 6, C-reactive protein (CRP) and D-dimer were related to hospitalization rates, rates of severe illness, and mortality rates following COVID-19 infection.^[7–11]^ Cancer patients with COVID-19 have been found to be at higher risk of hospitalization, requiring oxygen support, being admitted to intensive care units, and mortality rate.^[12]^ However, few studies have assessed the role of CRP in cancer patients with COVID-19 infection.^[13–15]^

Therefore, this cross-sectional study aims to assess the association between serum CRP levels and COVID-19 pneumonia in cancer patients infected with the Omicron variants from December 8, 2022 to February 1, 2023 in China.

## 2. Methods

### 2.1. Design and patients

This study followed the Strengthening the Reporting of Observational Studies in Epidemiology (STROBE) reporting guideline.^[16]^ This retrospective cross-sectional study assessed hospitalized patients with solid tumors who were diagnosed with Omicron variant COVID-19 between December 8, 2022 and February 1, 2023 during the Omicron variant epidemic at 2 institutions in Jiangxi Province, China, namely The Second Affiliated Hospital of Nanchang University and The First People Hospital of Fuzhou. The inclusion criteria were patients of all age groups with pathologically confirmed malignant tumors and diagnosed with Omicron variant COVID-19 by reverse transcriptase-polymerase chain reaction (RT-PCR) using nasopharyngeal swabs. Exclusion criteria were hematologic tumors and cases diagnosed only on the basis of rapid antigen testing. All hospitalized patients have received appropriate treatment and care since admission for COVID-19 according to NCCN clinical practice guidelines in oncology prevention and treatment of cancer-related infections (NCCN Guidelines),^[6]^ such as chest computer tomography (CT) scans, routine blood tests, biochemical tests, inflammation-related biomarkers. The institutional ethics committees of the Nanchang University reviewed and approved the study protocol.

### 2.2. Data collection

The clinical data regarding patient characteristics, including patient age, sex, the tumor stages, history of previous treatment and comorbidities were obtained from the electronic medical records. The laboratory examinations included routine blood tests, biochemical tests, inflammation-related biomarkers, and coagulation function. All laboratory parameters were performed on the 24 hours of hospital admission. The TNM staging of solid tumors was based on the 8th edition of the AJCC staging system. Patients were definitely diagnosed with COVID-19 pneumonia by chest CT scans. Patients with COVID-19 Severity were categorized as mild, moderate, severe, and critical based on NCCN Guidelines of cancer-related infections.^[6]^

### 2.3. Statistical analysis

All data were analyzed using R software. Categorical variables were presented as frequency and percentage, while continuous variables were expressed as mean ± standard deviation. The enrolled population was divided into 3 equal parts according to CRP, the low CRP group reference values 3.0 ± 2.0 mg/L, the high CRP group reference values 137.5 ± 62.5 mg/L. Independent sample t-tests and analysis of variance (ANOVA) were used to compare groups. Univariate and multivariate logistic regression analyses were performed to assess the association between serum CRP and the incidence of COVID-19 pneumonia, Model I: no covariates were adjusted; Model II: sex and targeted therapy before COVID-19 infection were adjusted; Model III: sex, targeted therapy and anti-angiogenic therapy before COVID-19 infection were adjusted, and the OR with 95% confidence intervals (CI) were reported. Statistical significance was set at *P* < .05.

## 3. Results

### 3.1. Patient characteristics

**Figure 1.**
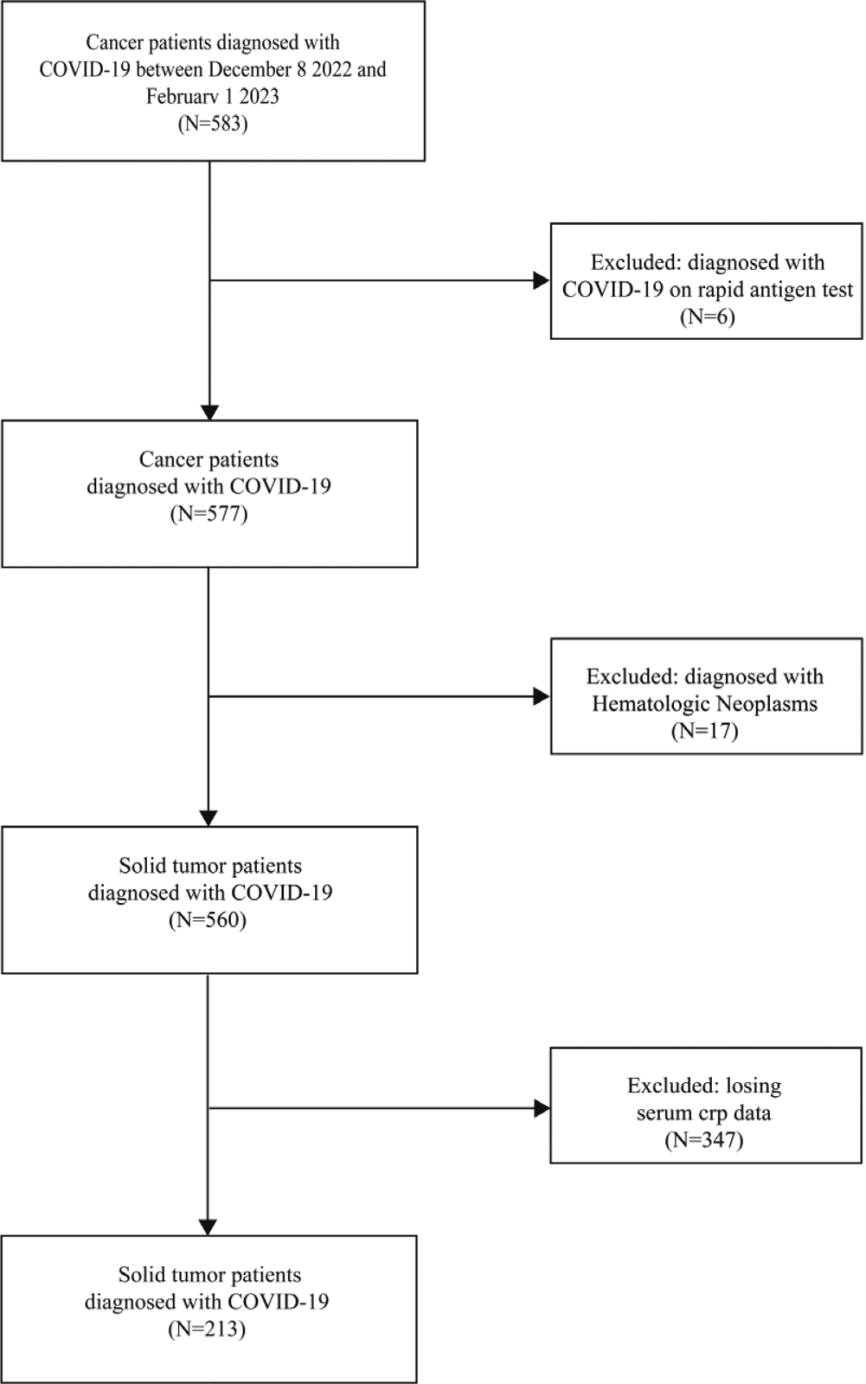
Flowchart of patient selection.

We conducted a retrospective analysis of 213 patients from the Department of Oncology at the Second Affiliated Hospital of Nanchang University and the First People Hospital of Fuzhou between December 8, 2022 and February 1, 2023. The flowchart illustrating the selection process is shown in Figure 1. Table 1 summarized the baseline demographic, clinical, and biochemical characteristics of the included patients. Of these patients, 41 (19.2%) had a history of comorbidities, and 130 (61%) patients were diagnosed with lung cancer, with the majority of stage IV patients (65.3%).

**Table 1:** Baseline characteristics of all enrolled patients.

Characteristics	CRP Low N = 71	CRP Middle N = 70	CRP High N = 72	*P* value
Age (yr)				.067
<70	55 (77.5%)	42 (60.0%)	46 (63.9%)	
≥70	16 (22.5%)	28 (40.0%)	26 (36.1%)	
Sex				.344
Female	25 (35.2%)	17 (24.3%)	20 (27.8%)	
Male	46 (64.8%)	53 (75.7%)	52 (72.2%)	
Comorbidities				.113
No	62 (87.3%)	57 (81.4%)	53 (73.6%)	
Yes	9 (12.7%)	13 (18.6%)	19 (26.4%)	
Tumor types				.073
Non- Lung cancer	20 (28.2%)	31 (44.3%)	32 (44.4%)	
Lung cancer	51 (71.8%)	39 (55.7%)	40 (55.6%)	
TNM stage				.056
I-III	18 (25.4%)	24 (34.3%)	32 (44.4%)	
IV	53 (74.6%)	46 (65.7%)	40 (55.6%)	
WBC (10^9^/L)	5.4 ± 2.4	5.5 ± 2.7	7.2 ± 4.7	.011
Neutrophil(10^9^/L)	3.6 ± 2.7	4.0 ± 2.4	6.7 ± 8.4	<.001
Lymphocyte(10^9^/L)	1.2 ± 1.2	0.9 ± 0.5	0.9 ± 1.0	.008
NLR	3.9 ± 3.4	6.1 ± 7.3	10.6 ± 14.0	<.001
CRP (mg/L)	3.0 ± 2.0	23.2 ± 12.2	137.5 ± 62.5	<.001
Chemotherapy				.286
No	36 (51.4%)	40 (61.5%)	35 (48.6%)	
Yes	34 (48.6%)	25 (38.5%)	37 (51.4%)	
Immunotherapy				.133
No	34 (48.6%)	37 (56.9%)	47 (65.3%)	
Yes	36 (51.4%)	28 (43.1%)	25 (34.7%)	
Radiotherapy				.359
No	62 (89.9%)	54 (83.1%)	65 (90.3%)	
Yes	7 (10.1%)	11 (16.9%)	7 (9.7%)	
Anti-angiogenic therapy				.217
No	57 (81.4%)	51 (78.5%)	50 (69.4%)	
Yes	13 (18.6%)	14 (21.5%)	22 (30.6%)	
Targeted therapy				.413
No	52 (74.3%)	48 (73.8%)	47 (65.3%)	
Yes	18 (25.7%)	17 (26.2%)	25 (34.7%)	
COVID-19 pneumonia				<.001
No	63 (88.7%)	42 (60.0%)	22 (30.6%)	
Yes	8 (11.3%)	28 (40.0%)	50 (69.4%)	
Severe COVID-19				<.001
No	71 (100.0%)	68 (97.1%)	51 (70.8%)	
Yes	0 (0.0%)	2 (2.9%)	21 (29.2%)	

Comorbidities included chronic obstructive pulmonary disease, hypertension and cardiovascular disease. The patient previous treatment history before COVID-19 infection included chemotherapy, radiotherapy, immunotherapy, targeted therapy and Anti-angiogenic therapy.

ICU = Intensive Care Unit, CRP = C-reactive protein, NLR = leukocyte to lymphocyte ratio, WBC = white blood cell.

### 3.2. COVID-19 pneumonia profiles

Overall, 86 (40.4%) patients developed Omicron variants COVID-19 pneumonia. There was 8 (11.3%) and 28 (40.0%) patients with COVID-19 pneumonia in the low and medium CRP groups, respectively. In the high CRP group, 50 (69.4%) patients experienced COVID-19 pneumonia. Furthermore, no patients with severe pneumonia were found in the low CRP group, 2 (2.9%) patients with severe pneumonia in the middle CRP group, and 21 (29.17%) patients in the high CRP group.

### 3.3. Association between CRP and COVID-19 pneumonia

**Figure 2.**
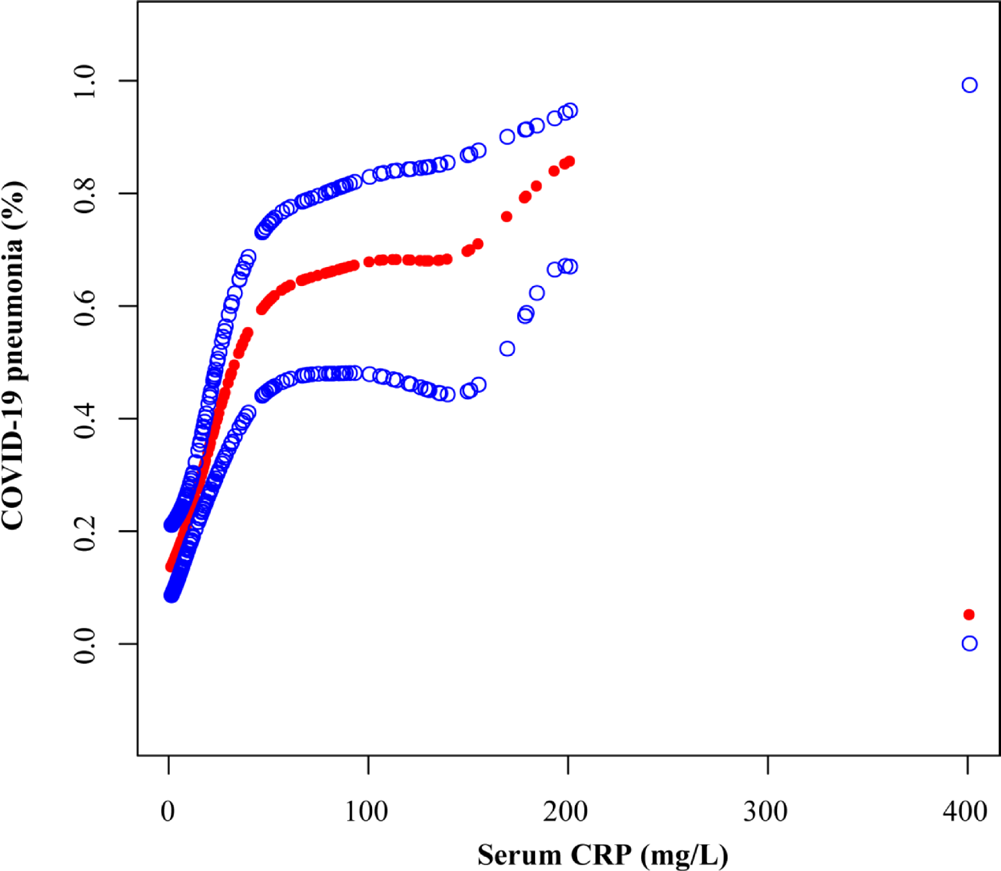
Association between the serum CRP and the incidence of COVID-19 pneumonia in cancer patients. CRP = C-reactive protein.

As shown in Table 2, in our univariate analysis of the entire cohort, high CRP was found to be an unfavorable factor of COVID-19 pneumonia (OR = 17.9, 95%CI: 7.3, 43.6; *P <* .001). In addition, high neutrophil-to-lymphocyte ratio (NLR) (OR = 1.1, 95%CI: 1.0, 1.2; *P <* .001) and age ≥ 70 (OR = 2.4, 95%CI: 1.3, 4.2; *P* = .004) were associated with higher incidence rate of COVID-19 pneumonia. High lymphocyte count (OR = 0.3, 95%CI: 0.1, 0.5; *P <* .001) and lung cancer (OR = 0.3, 95%CI: 0.2, 0.6; *P <* .001) had a lower incidence rate of COVID-19 pneumonia. In the multivariate analysis, the incidence rate of COVID-19 pneumonia was associated with CRP, age, and tumor type (Table 3). After adjustment of gender, targeted therapy and anti-angiogenic therapy, the incidence of COVID-19 pneumonia remained positively associated only with serum CRP (OR = 9.8, 95%CI: 2.2, 43.8; *P* = .003) and was negatively associated with lung cancer (OR = 0.3, 95%CI: 0.1, 0.7; *P* = .006). Nevertheless, lymphocyte count (OR = 0.6, 95%CI:0.3,1.2; *P* = .146), NLR (OR = 1.0, 95%CI:1.0, 1.1; *P* = .442), age (OR = 2.1, 95%CI:1.0, 4.6; *P* = .059), History of comorbidities (OR = 1.3, 95%CI:0.5, 3.2; *P* = .638), and tumor stage (OR = 1.2, 95%CI:0.5, 2.7; *P* = .662) were not significantly associated with COVID-19 pneumonia. Through the multivariate logistic regression model and smooth curve fitting, we further observed that the relationship between serum CRP and Omicron variants COVID-19 pneumonia was non-linear (Fig. 2).

**Table 2:** Univariate analysis of factors associated with COVID- 19 pneumonia.

	COVID- 19 pneumonia	
	OR (95%CI)	*P* value
Age (yr)		.004
<70	1	
≥70	2.4 (1.3, 4.2)	
Sex		.532
Female	1	
Male	1.2 (0.7, 2.2)	
Comorbidities		.056
No	1	
Yes	2.0 (1.0, 3.9)	
Tumor types		<.001
Non- Lung cancer	1	
Lung cancer	0.3 (0.2, 0.6)	
TNM stage		.134
I-III	1	
IV	0.6 (0.4, 1.1)	
WBC (10^9^/L)	1.0 (0.9, 1.1)	.636
Neutrophil(10^9^/L)	1.0 (1.0, 1.1)	.823
Lymphocyte(10^9^/L)	0.3 (0.1, 0.5)	<.001
NLR	1.1 (1.0, 1.2)	<.001
CRP (mg/L)	1.0 (1.0, 1.0)	<.001
CRP groups		
Low	1	
Middle	5.2 (2.2, 12.6)	<.001
High	17.9 (7.3, 43.6)	<.001
Chemotherapy		.402
No	1	
Yes	0.8 (0.5, 1.4)	
Immunotherapy		.144
No	1	
Yes	0.7 (0.4, 1.2)	
Radiotherapy		.343
No	1	
Yes	0.7 (0.3, 1.6)	
Anti-angiogenic therapy		.172
No	1	
Yes	1.6 (0.8, 3.0)	
Targeted therapy		.606
No	1	
Yes	1.2 (0.6, 2.2)	

Comorbidities included chronic obstructive pulmonary disease, hypertension and cardiovascular disease. The patient previous treatment history before COVID-19 infection included chemotherapy, radiotherapy, immunotherapy, targeted therapy and anti-angiogenic therapy.

CI = confidence interval, CRP = C-reactive protein, NLR = leukocyte to lymphocyte ratio, OR = odds ratio, WBC = white blood cell,

**Table 3:** Multivariate analysis of factors associated with COVID-19 pneumonia.

	COVID- 19 pneumonia					
	Model I OR(95%CI)	*P* value	Model II OR(95%CI)	*P* value	Model III OR(95%CI)	*P* value
Age (yr)		.044		.054		.059
<70	1		1		1	
≥70	2.2 (1.0, 4.6)		2.2 (1.0, 4.7)		2.1 (1.0, 4.6)	
Comorbidities		.540		.604		.638
No	1		1		1	
Yes	1.3 (0.5, 3.3)		1.3 (0.5, 3.3)		1.3 (0.5, 3.2)	
Tumor types		.004				.006
Non- Lung cancer	1		1	.006	1	
Lung cancer	0.3 (0.2, 0.7)		0.3 (0.1, 0.7)		0.3 (0.1, 0.7)	
TNM stage		.771		.611		.662
I-III	1		1		1	
IV	1.1 (0.5, 2.4)		1.2 (0.5, 2.8)		1.2 (0.5, 2.7)	
Lymphocyte(10^9^/L)	0.6 (0.3, 1.2)	.125	0.6 (0.3, 1.2)	.148	0.6 (0.3, 1.2)	.146
NLR	1.0 (1.0, 1.1)	.378	1.0 (1.0, 1.1)	.428	1.0 (1.0, 1.1)	.442
CRP (mg/L)	1.0 (1.0, 1.0)	.397	1.0 (1.0, 1.0)	.391	1.0 (1.0, 1.0)	.411
CRP groups						
Low	1				1	
Middle	3.5 (1.4, 9.0)	.009	4.3 (1.6, 11.7)	.004	4.3 (1.6, 11.8)	.004
High	8.5 (2.0, 36.7)	.004	9.8 (2.2, 43.5)	.003	9.8 (2.2, 43.8)	.003

Comorbidities including patients on mechanical ventilation and extracorporeal mechanical oxygenation (ECMO) and end organ dysfunction. ModelI: no covariates were adjusted; model II: sex and targeted therapy before COVID-19 infection were adjusted; model III: sex, targeted therapy and anti-angiogenic therapy before COVID-19 infection were adjusted.

CI = confidence interval, CRP = C-reactive protein, NLR = leukocyte to lymphocyte ratio, OR = odds ratio.

## 4. Discussion

This cross-sectional analysis of data from December 8, 2022 to February 1, 2023 found that the association between serum CRP and Omicron variant COVID-19 pneumonia in cancer patients from China, which has certain guiding significance for the treatment and prognosis of cancer patients after contracting COVID-19. This study is the first cross-sectional study analyzing the association between serum CRP in cancer patients and Omicron variant COVID-19 pneumonia since the change in prevention and control policy of China.

Some retrospective studies shows Omicron has a much lower risk of severe outcomes after SARS-CoV-2 infection than Delta.^[1,17,18]^ A study from England reported that for Omicron BA.2 subvariant, the critical illness rate has dropped to 0.4% and the mortality rate has dropped to 0.3%.^[19]^ These studies are consistent with our findings, which shows that few patients had severe Omicron variants COVID-19 pneumonia. CRP is an acute-phase protein whose concentration may increase over 1000-fold in inflammatory states.^[20]^ Correlations between CRP concentrations and severity of COVID-19 disease have been reported in multiple series.^[21–25]^ In a prior study of 2601 patients with COVID-19, CRP concentrations above the median value (108 mg/L) were associated with critical illness (47.6% vs 25.9%; OR 2.83, 95% CI 2.37–3.37), and mortality (32.2% vs 17.8%; OR 2.59, 95% CI 2.11–3.18), compared with CRP below the median.^[21]^ A meta-analysis found that compared to the severe group, the non-severe group of COVID-19 patients had lower levels of CRP by analyzing 16 studies including 3962 COVID-19 patients.^[25]^ On the contrary, A retrospective study of 85 patients with COVID-19 found that CRP levels are not related to severe COVID-19 pathology or disease severity.^[26]^ Gao and colleagues found that there is no direct relationship between the severity of COVID-19 symptoms and the circulating levels of IL-2, IL-4, TNF-α, IFN-γ, and CRP.^[27]^ However, the sample in these studies are relatively small and selection bias may occur. In contrast, our study found that high CRP levels is significantly associated with the incidence of COVID-19 pneumonia in cancer patients.

Although some studies have reported on the association between CRP concentration and disease severity. Omicron B.1.1.529 variant of SARS-CoV-2 has been reported as less severe, but more transmissible than previous variants.^[1,17,18]^ The proportion of severe COVID-19 is much higher in vulnerable populations such as cancer patients.^[15,27,28]^ Zhou Y and colleagues recruited 103 COVID-19 cancer patients and found that NLR and CRP were important risk factors for death and developed a line chart based on these 2 factors to predict the probability of death, with an AUC of 0.918.^[29]^ A multicenter retrospective study of 84 gynecological cancer patients found that CRP levels were significantly elevated in patients with severe infections (median 142.0 mg/L, IQR 62.4–217.1 vs median 62.3 mg/L, IQR 13.0–159.1; *P* = .02).^[30]^ Most of these studies focused on Alpha strains. In this study, we did not analyze the association between CRP and severe pneumonia due to the low critical illness rate and mortality of Omicron.

In addition, we also found through multivariate analysis that lung cancer patients were less likely to develop pneumonia after COVID-19 infection (OR = 0.3, 95%CI: 0.1, 0.7; *P* = .006). This is contrary to some research results.^[28,31,32]^ This may be related to the atypical radiological manifestations of COVID-19 pneumonia, which are difficult to distinguish from obstructive pneumonia, immune-related pneumonia, and radiation-induced pneumonia.^[33]^

Some studies reported that other inflammatory markers are also associated with adverse outcomes in COVID-19, such as NLR, platelet/lymphocyte ratio, serum procalcitonin, ferritin, D-dimer, LDH, IL-6 etc.^[34–37]^ Conversely, we analyzed other hematological indicators and found that lymphocyte count (*P* = .146) and neutrophil/lymphocyte ratio (*P* = .442) were not significantly correlated with occurrence of COVID-19 pneumonia. In addition, this study excluded patients with hematological malignancies. However, a retrospective study found that compared to patients with solid malignant tumors, patients with hematological diseases had an increased risk of severe COVID-19 outcomes and death (including within 7 days) (OR: 2.16, 95% CI: 1.18–3.95).^[38]^ Molins B et al also explored the ability of different CRP subtypes to predict the prognosis of COVID-19 and found that CRP levels above 4000 ng/mL (OR: 4.551, 95% CI: 1.329–15.58) could be used as a marker for the clinical severity of COVID-19.^[7]^ Ultimately, CRP is an applicable biomarker of COVID-19 pneumonia since it is inexpensive and widely available at most medical institutions.

The study has certain limitations. Firstly, being a retrospective study, it inevitably carries inherent selection biases. Secondly, the Charlson Comorbidity Index has been identified as an independent prognostic indicator for COVID-19 patients, especially during the Omicron dominance period.^[39,40]^ In our study, we did not independently assess the prognostic significance of Charlson Comorbidity Index following COVID-19 Omicron infection in cancer patients. However, we did analyze the impact of complications (such as chronic obstructive pulmonary disease, hypertension, cardiovascular disease, etc) on COVID-19 outcomes. Thirdly, the sample size in this study was relatively limited, and a larger sample might unveil associations between serum CRP levels and severe COVID-19 pneumonia in cancer patients. Finally, this study specifically focused on the Omicron variants (BA.2 and BA.5). As SARS-CoV-2 continues to evolve, new strains may emerge in the future.^[41]^ Hence, exploratory studies are imperative to investigate the molecular mechanisms and epidemiological characteristics of these new variants. Additionally, the association between cancer treatment-related toxic side effects and COVID-19 infection remains unknown and necessitates further prospective studies.

## 5. Conclusion

In conclusion, this study found that cancer patients with higher serum CRP were more likely to suffering Omicron variant COVID- 19 pneumonia. Therefore, chest CT images are recommended when cancer patients with Omicron variant COVID- 19 diseases have high CRP levels.

## Acknowledgments

We sincerely thank the patients treated in our hospitals.

## Author contributions

**Conceptualization:** Zhimin Zeng, Anwen Liu, Yanqing He.

**Data curation:** Kaijun Che, Chen Hong.

**Formal analysis:** Kaijun Che, Chen Hong.

**Investigation:** Kaijun Che, Chen Hong, Yanqing He.

**Methodology:** Kaijun Che, Chen Hong, Duanyang Peng.

**Project administration:** Duanyang Peng, Anwen Liu.

**Resources:** Anwen Liu, Yanqing He.

**Software:** Zhimin Zeng, Duanyang Peng.

**Supervision:** Zhimin Zeng, Duanyang Peng, Anwen Liu, Yanqing He.

**Validation:** Zhimin Zeng, Anwen Liu, Yanqing He.

**Visualization:** Zhimin Zeng, Anwen Liu, Yanqing He.

**Writing – original draft:** Kaijun Che, Chen Hong.

**Writing – review & editing:** Kaijun Che, Chen Hong.
